# ICU Admission, Invasive Mechanical Ventilation, and Mortality among Children and Adolescents Hospitalized for COVID-19 in a Private Healthcare System

**DOI:** 10.1155/2023/1698407

**Published:** 2023-02-23

**Authors:** Maria da Gloria Cruvinel Horta, Geraldo Jose Coelho Ribeiro, Nelson Otavio Beltrao Campos, Douglas Ribeiro de Oliveira, Lelia Maria de Almeida Carvalho, Karina de Castro Zocrato, Daniel Pitchon dos Reis, Mariana Ribeiro Fernandes, Ricardo Mesquita Camelo, Fernando Martin Biscione, Silvana Marcia Bruschi Kelles

**Affiliations:** ^1^Research and Health Care Management (Gerencia de Pesquisa e Atençao a Saude, GPAS), Unimed-BH, Belo Horizonte, Brazil; ^2^Health Data Science Management (Gerencia de Ciencia de Dados em Saude, GCDS), Unimed-BH, Belo Horizonte, Brazil; ^3^Faculty of Medicine, Universidade Federal de Minas Gerais, Belo Horizonte, Brazil; ^4^Faculty of Medicine, Pontificia Universidade Catolica de Minas Gerais, Belo Horizonte, Brazil

## Abstract

**Aim:**

The COVID-19 pandemic devastated healthcare around the world. Data about the COVID-19 outcomes among young people are still scarce. We aim to identify factors associated with the composite outcome among children and adolescents hospitalized due to COVID-19.

**Methods:**

We performed a search in the database of a large Brazilian private healthcare system. Insured people aged 21 years or younger who were hospitalized due to COVID-19 from Feb/28th/2020 to Nov/1st/2021 were included. The primary endpoint was the composite outcome consisting of ICU admission, need for invasive mechanical ventilation, or death.

**Results:**

We evaluated 199 patients who had an index hospitalization due to COVID-19. The median monthly rate of index hospitalization was 2.7 (interquartile range [IQR], 1.6-3.9) per 100,000 clients aged 21 years or less. The median age of the patients was 4.5 years (IQR, 1.4-14.1). At the index hospitalization, the composite outcome rate was 26.6%. The composite outcome was associated with all the previous coexisting morbidities evaluated. The median follow-up was 249.0 days (IQR, 152.0-438.5). There were 27 readmissions (16 patients) within 30 days after the discharge.

**Conclusions:**

In conclusion, hospitalized children and adolescents had a composite outcome rate of 26.6% at the index hospitalization. Having previous chronic morbidity was associated with the composite.

## 1. Introduction

The severe acute respiratory syndrome coronavirus type 2 (SARS-CoV-2) pandemic has devastated populations around the world since the last days of 2019 [1]. The SARS-CoV-2 disease (COVID-19) is characterized by respiratory signs and symptoms, but other organs or systems may be involved, including neurological manifestations and thrombosis [2]. However, one-fifth up to one-third of infected people may be asymptomatic [3, 4]. By Nov/1^st^/2021, there were 247.59 million confirmed COVID-19 cases worldwide, of whom 5.01 million were deceased due to the disease (https://ourworldindata.org/coronavirus). In Brazil, by the same date, there were 21.82 million confirmed COVID-19 cases, of whom 608,185 were deceased (https://ourworldindata.org/coronavirus).

Despite a recent meta-analysis having reported a rate of 32.1% of asymptomatic infections among children and adolescents [4], they develop symptomatic COVID-19. The spectrum of the disease signs and symptoms varies from fever, cough, and nasal symptoms to respiratory distress and arrhythmia [5–7]. Radiological findings (e.g., ground-glass opacities) are also present [5–7]. Notwithstanding, they seem to have a milder disease course with a better outcome than the adults [5, 8, 9]. Therefore, a low rate of children and adolescents who develop COVID-19 needs to be hospitalized [10, 11]. Overall, outpatient need for admission to the intensive care unit (ICU) and deaths (fatality rate) range about 4%-11% and 0.0%-2.4%, respectively [6, 12–15].

A routinely collected nationwide Brazilian surveillance database recently described the outcome of 11,613 children and adolescents 20 years or younger hospitalized in both public and private hospitals, with laboratory-confirmed SARS-CoV-2 infection, from Feb/16^th^/2020 to Jan/9^th^/2021 [16]. The authors reported an overall fatality rate of 7.6% [16]. Based on the Brazilian national system of mortality information, it was noted that, among people aged 0 to 19 years, the number of observed deaths was lower than expected during the pandemic period (Dec/29^th^/2019 to Jan/2^nd^/2021) related to the years 2015 until 2019 [17]. Unfortunately, public and private healthcare provisions for COVID-19 may be different in Brazil, due to socioeconomical inequalities and social vulnerability [16, 18, 19]. Besides that, there is a paucity of data about the outcomes of children and adolescents hospitalized due to COVID-19 in Brazilian private hospitals. This study was aimed at identifying factors associated with the composite outcome of ICU admission, need for invasive mechanical ventilation, or death among children and adolescents hospitalized due to COVID-19 and covered by a large private healthcare system located in the center of a southeastern Brazilian state.

## 2. Methods

### 2.1. Study Design and Ethical Aspects

We conducted a retrospective cohort study based on administrative data, according to the Brazilian Legislation on Research on Humans. It was approved by the Committee on Ethics on Research of the Faculdade de Ciências Médicas de Minas Gerais (CAAE 34405320.4.0000.5134, on Jul/23^th^/2020). The consent form was waived due to the study design since no intervention would be performed. Only anonymized data were collected. Financial support was fully provided by the healthcare maintenance organization (HMO).

### 2.2. Setting

The study was based on administrative data from a large southeastern Brazilian HMO. It operates in the capital of the state of Minas Gerais (Belo Horizonte) and other 33 surrounding municipalities. It provides healthcare for more than 1.3 million insured people with a coverage area of 11,700.00 km [2] and 5.1 million inhabitants. It is the largest medical cooperative of the complementary healthcare system in Brazil which provides patient healthcare assistance at outpatient medical clinics and offices, home care, telemedicine support, prehospital ambulance system, and 25 tertiary/quaternary hospitals.

### 2.3. Data Storage, Sources, Audition, and Protection

The HMO uses two own-service database storage systems: Data Lake, from the Amazon Web Services (https://aws.amazon.com/lake-formation/), and the Data Warehouse, from the Oracle Warehouse Builder (https://www.oracle.com/autonomous-database/autonomous-data-warehouse/). A Data Lake is a centralized repository that allows storage of all structured and unstructured data at any scale. A Data Warehouse is a data management system designed to enable and support business intelligence activities, especially analytics. Both systems are intended to perform queries and analyses and often contain large amounts of historical data.

The HMO database is updated by both healthcare providers and administrative personnel, from each healthcare service. Data are audited by an accredited team from each healthcare service and centrally. Data are individualized and anonymized by an identification code. The demographic and clinical data, such as ICD-10, outpatient medical appointments, hospitalizations, surgeries, dialysis, and procedures (e.g., invasive mechanical ventilation and hemodialysis), are included. These data are routinely updated to provide the spectrum of complex chronic condition classification of each patient, both children and adults [20–22]. Especially in the case of hospitalization, one Diagnosis-Related Group (DRG) was attributed to each admission [23]. DRGs provide guidelines to determine hospital reimbursement by many insurance providers. This encompasses several metrics designed to classify the healthcare resources based on diagnosis, prognosis, and various other factors [23]. Besides that, there is an audit of deaths in each hospital provider and centrally. Then, it is possible to follow the patient as long as he/she remains insured by the HMO.

Finally, restricted access is accomplished by the HMO to guarantee the protection of personal data. The HMO follows the recommendations of the Brazilian General Personal Data Protection Law (http://www.planalto.gov.br/ccivil_03/_ato2015-2018/2018/lei/l13709.htm). Data were extracted from the HMO database platform using an anonymized search.

### 2.4. Participants

We selected all the hospitalizations due to COVID-19 of patients insured by the HMO who were 21 years or below at the date of the index hospitalization [24]. The study period was from Feb/28^th^/2020 to Nov/1^st^/2021. We considered the index hospitalization due to COVID-19 if (i) the hospitalization was classified according to the International Classification of Disease-10^th^ revision (ICD-10) as U07.1, B34.2, or B97.2 and/or (ii) the patient had a positive real-time reverse transcriptase-polymerase chain reaction (RT-qPCR) for SARS-CoV-2 in the upper respiratory tract swab during the hospitalization, despite the main ICD-10. Hospitalization was a shared decision between the physician and the patient and/or his/her guardian. Patients hospitalized at the HMO's services but who were insured by other companies were excluded, due to the lack of previous and follow-up data. After performing the database search, we also reviewed the newborns' hospitalizations, to ensure that their diagnoses were COVID-19 and not their mother's. After revision, admitted newborns without a conclusive inclusion criterium were excluded. No patient was still in hospitalization at the end of the study period.

### 2.5. Variables

The follow-up period was calculated from the index hospitalization date until one endpoint was reached: death, loss of insurance coverage, or end of the study (Nov/1^st^/2021). Age at the index admission, sex, need for hemodialysis, and dates of death or loss of the insurance coverage were extracted from the HMO's database. Length of stay was calculated from admission until discharge or death. Previously coexisting morbidities were obtained, according to the classification of complex chronic conditions [20–22]. Among the coexisting morbidities, only those attributed to 15 or more patients were evaluated. ICU admission, need for invasive mechanical ventilation, and need for hemodialysis were indicated at physician discretion. Due to hospital practices, patients 14 years old or more were treated in adult settings, while the youngest ones were admitted in pediatric settings. Readmissions to the hospital comprised—and were undifferentiated—of noncritical care wards or the ICU. Readmissions were assessed according to three different definitions: (i) readmissions along the study follow-up period (from the index hospitalization until endpoint), (ii) readmissions within 30 days of the discharge from the index hospitalization, and (iii) readmissions related to COVID-19, which follow the same definitions as stated above for the index hospitalization (i.e., ICD-10 related to COVID-19 and/or positive RT-qPCR for SARS-CoV-2 during hospitalization).

### 2.6. Statistical Methods

The primary endpoint was the composite which comprised any of the following outcomes: ICU admission, need for invasive mechanical ventilation, or death during the index hospitalization. We used this composite as an endpoint based on two assumptions: firstly, ICU admission rates for COVID-19-positive children and adolescents are common, and they are often associated with mechanical ventilation; in addition, deaths related to COVID-19 among children and adolescents are not negligible [11, 12, 15, 25]. However, the unit of analysis was the patient. We established two groups: the composite negative (the patient had none of the composite items) and the composite positive (the patient had at least one outcome of the composite). The secondary outcome was the need for readmission.

We used the R Statistics and R Studio-4.1.2 for Windows (32-/64-bit) to analyze the data. Categorical variables were described as absolute and relative frequency and continuous variables as median and interquartile ranges. The differences between the groups were inferred by the odds ratio (OR) and 95% confidence intervals, followed by Fisher's exact test (frequencies), or Mann–Whitney *U* test (medians with interquartile range [IQR]). The level of significance was established as *p* < 0.05.

## 3. Results

**Figure 1:**
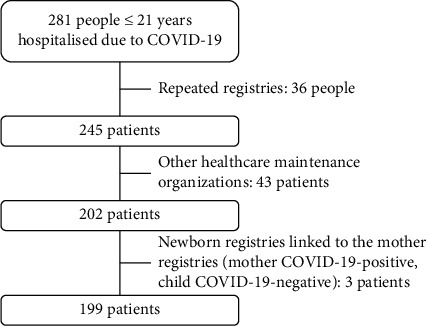
Study flowchart.

The database search retrieved 281people ≤ 21 years who were hospitalized due to COVID-19 (Figure 1). We excluded 36 repeated registries, 43 patients who were not insured by the current HMO, and 3 newborns who were COVID-19 negative but whose hospitalization registries were linked to their COVID-19-positive mothers' registries. The remaining 199 patients comprised 271 hospitalizations which involved 199 (73.4%) index hospitalizations due to COVID-19 and 72 (26.6%) readmissions during the study period, among 43 patients (Table 1). No patient waited longer than 24 h to be hospitalized after the admission requirement was signed by the physician. In addition, no child nor adolescent died while waiting for a hospital bed.

The mean (±standard deviation) monthly number of HMO-registered people aged 21 years or younger remained stable throughout the study period (325,254 ± 8253 people/month). The index hospitalizations occurred throughout the study period, except in Oct/2021 when no admission was described. The median monthly rate of index hospitalizations was 2.7 (IQR, 1.6-3.9) per 100,000 clients aged 21 years or less.

Demographic and clinical data are presented in Table 1. The median age of the patients at the index hospitalization was 4.5 years (IQR, 1.4-14.1), of whom the majority (105/199; 52.8%) were 5 years or younger. The median length of stay of the index hospitalization was 3.0 days (IQR, 2.0-7.0). There were 3 (1.5%) deaths and 12 (6.0%) patients lost to follow-up due to insurance coverage. The median follow-up since the index admission was 249.0 days (IQR, 152.0-438.5). Two (1.0%) patients started hemodialysis at the index hospitalization. Seventy-three (36.7%) patients reported no previous coexisting morbidity. Among the reported coexisting morbidities, the most prevalent was asthma (72/199; 36.2%). Readmissions were evaluated for 196 patients since 3 (1.5%) died during the index hospitalization. Sixteen (8.2%) patients had 27 (10.0%) readmissions within 30 days after the discharge of the index hospitalization, with a median of 10.5 days (IQR, 5.3-14.3) between the discharge and the first readmission. Twenty-eight (10.3%) COVID-19-related readmissions were reported for 22 (11.2%) patients, during the study period, in a median of 47.5 days (IQR, 15.0-64.5), between the discharge and the first readmission.

During the index hospitalization, the composite outcome was reported for 53 (26.6%) patients (Table 2). ICU admissions, need for invasive mechanical ventilation, or death was reported for 52 (23.1%), 29 (14.6%), and 3 (1.5%) patients, respectively. After the discharge of the index hospitalization, the composite outcome was reported for 6 (3.1%) patients. During the whole study period, the composite outcome was reported for 58 (29.2%) patients.

The variables associated with the composite outcome during the index hospitalization are described in Table 3. The length of stay of patients who presented the composite (10.0 days [IQR, 6.0-17.0]) was longer than the length of stay of those did not have the composite (2.0 days [2.0-4.0]) (*p* ≤ 0.001). Having previously coexisting morbidity was associated with the composite: one morbidity resulted in an OR 4.08 (95% CI, 1.40-13.64) (*p* = 0.005) and two or more morbidities resulted in an OR 9.96 (95% CI, 3.62-32.27) (*p* ≤ 0.001). All the reported coexisting morbidities were associated with the composite outcome. The highest association was found for the presence of gastrointestinal diseases: OR 9.24 (95% CI, 3.34-28.47) (*p* ≤ 0.001).

## 4. Discussion

The current study showed that hospitalized children and adolescents have a composite outcome (ICU admission, need for invasive mechanical ventilation, or death) rate of 26.6% during the index hospitalization. We found that 23.1% needed admission to the ICU, which is similar to a Brazilian database (23.8%) [16], but much higher than the rate reported by a North American database (6.5%) [11]. However, we found the opposite for the fatality rate: we reported 1.5%, similar to the North American study (2.2%) [16], but lower than the Brazilian one (12.1%) [11]. Such discrepancies may reflect the socioeconomical inequalities and social vulnerability of the Brazilian population facing healthcare provision during the pandemic [18, 19]. As described by the authors of the previously cited Brazilian study [16], among 8,341 children and adolescents who were not admitted to the ICU, 170 required invasive mechanical ventilation, and 85 (50.0%) died. Of the 2,759 children and adolescents who were admitted to the ICU, 991 required invasive mechanical ventilation and 391 died (39.5%). The lack of appropriate intensive care assistance may have contributed to the higher fatality rate among invasive mechanically ventilated people admitted to the non-ICU wards compared to those who were treated at the ICU. In our study, the low waiting time from hospitalization request until admission to a hospital bed may have resulted in the low fatality rate in the ICU during index hospitalizations.

Having previously chronic morbidity was associated with the occurrence of the composite in our analysis. This corroborates a meta-analysis that showed that, compared to children without underlying diseases, children with comorbidities had a higher relative risk ratio of developing severe COVID-19 and COVID-19-associated mortality [26]. In the United States of America, the demographic and clinical factors associated with deaths due to COVID-19 among people aged 21 years or younger included having one or more underlying conditions [24]. A multinational European cohort study showed that the presence of any preexisting medical condition was associated with ICU admissions among children and adolescents, especially chronic pulmonary disease [25]. In Brazil, having this previous morbidity was associated with both invasive mechanical ventilation and in-hospital death [16, 27].

All the evaluated preexisting conditions were associated with the composite outcome. The highest OR was found for gastrointestinal disease, and the lowest was found for asthma. Although we know the gastrointestinal tract may be affected by the SARS-CoV-2 [2], we could not find an explanation for this result. We believe the small number of patients may have impacted the statistical analysis.

### 4.1. Limitations

The study has limitations. Firstly, it is a retrospective study that was developed during a burst of a new severe disease. Although the multilevel audit process guarantees that the data are valid, both the diagnosis access at the beginning of the COVID-19 pandemic and the notification of some COVID-19-related conditions throughout the follow-up period may have been compromised. In particular, we could not access the pediatric multisystem inflammatory syndrome (PMIS) using the administrative data since the condition did not have an ICD-10 since the beginning of the study [28, 29]. However, it is a very rare disease, and we may have lost a few cases [28, 29]. Further analysis of our group will evaluate both outpatient and inpatient children and adolescents with COVID-19 and search their registries to identify PMIS. Secondly, since children and adolescents have no or milder symptomatology [5, 8, 9], the hospitalization may occur during a phase in which the SARS-CoV-2 cannot be identified, resulting in a false negative case [30]. Besides that, we did not consider the serological results as a COVID-19 criterium, due to its lower accuracy compared to the RT-qPCR [30]. Finally, patients who were admitted to the public or at other private healthcare systems were not enrolled in our analysis.

## 5. Conclusion

In conclusion, hospitalized children and adolescents showed a composite outcome rate of 26.6%, at the index hospitalization. Having previous chronic morbidity, especially gastrointestinal diseases, was associated with ICU admission, need for invasive mechanical ventilation, or death.

## Figures and Tables

**Table 1:** Demographic and clinical data of patients hospitalized due to COVID-19.

Characteristic	Total (*n* = 199)
Age at the index hospitalization, in years, median (IQR)	4.5 (1.4-14.1)
Male, in *n* (%)	99 (49.8%)
Hospital length of stay during the index hospitalization, in days, median (IQR)	3.0 (2.0-7.0)
Follow-up since the index hospitalization, in days, median (IQR)	249.0 (152.0-438.5)
Endpoint, in *n* (%)	
Active insurance/study deadline	184 (92.5%)
Loss of insurance	12 (6.0%)
Death	3 (1.5%)
Hemodialysis, in *n* (%)	
Previous hemodialysis	0 (0.0%)
Hemodialysis during or after the index hospitalization	2 (1.0%)
Number of morbidities, in *n* (%)	
0	73 (36.7%)
1	63 (31.7%)
≥2	63 (31.7%)
Morbidities, in *n* (%)	126 (63.3%)
Asthma	72 (36.2%)
Neurologic diseases	41 (20.6%)
Gastrointestinal diseases	24 (12.1%)
Congenital or chromosomal abnormalities	22 (11.1%)
Heart failure	19 (9.5%)
Obesity	15 (7.5%)
Chronic lung disease (except for asthma)	10 (5.0%)
Diabetes	7 (3.5%)
Readmissions (*n* = 196)	
Admissions during the follow-up period, in *n*	271
Readmissions during the follow-up period, in *n* (%)	72 (26.6%)
Number of people who were readmitted during the follow-up period, in *n* (%)	43 (21.9%)
Readmissions within 30 days after the discharge of the index hospitalization, in *n* (%)	27 (10.0%)
Number of people readmitted within 30 days after the discharge of the index hospitalization, in *n* (%)	16 (8.2%)
Time elapsed between the discharge of the index hospitalization and the first readmission within 30 days, in days, median (IQR)	10.5 (5.3-14.3)
Readmissions related to COVID-19 after the index hospitalization, in *n* (%)	28 (10.3%)
Number of people readmitted related to COVID-19 after the index hospitalization, in *n* (%)	22 (11.2%)
Time elapsed between the discharge of the index hospitalization and the first readmission related to COVID-19, in days, median (IQR)	47.5 (15.0-64.5)

COVID-19: coronavirus disease, 2019; IQR: interquartile range.

**Table 2:** Composite outcome of patients hospitalized due to COVID-19.

Characteristic	Total (*n* = 199)
During the index hospitalization	199
Admissions to the intensive care unit, in *n* (%)	52 (23.1%)
Need for invasive mechanical ventilation, in *n* (%)	29 (14.6%)
Deaths, in *n* (%)	3 (1.5%)
Composite outcome, in *n* (%)	53 (26.6%)
During the follow-up, after the index hospitalization	196
Admissions to the intensive care unit, in *n* (%)	6 (3.1%)
Need for invasive mechanical ventilation, in *n* (%)	2 (1.0%)
Deaths, in *n* (%)	0 (0.0%)
Composite outcome, in *n* (%)	6 (3.1%)
During the study period	199
Admissions to the intensive care unit, in *n* (%)	58 (29.2%)
Need for invasive mechanical ventilation, in *n* (%)	31 (15.6%)
Deaths, in *n* (%)	3 (1.5%)
Composite outcome, in *n* (%)	58 (29.2%)

**Table 3:** Variables associated with the composite outcome (intensive care unit admission, need for invasive mechanical ventilation, or death) during the index hospitalization due to COVID-19.

Characteristic	Patients			*p*	OR (95% CI)	*p*
	Total (*n* = 199)	Composite^−^ (*n* = 146)	Composite^+^ (*n* = 53)			
Age at index hospitalization, in years, median (IQR)	4.5 (1.4-14.1)	3.6 (1.0-14.8)	7.6 (1.9-10.9)	0.642		
Male, in *n* (%)	99 (49.8%)	74 (50.7%)	25 (47.2%)		0.87 (0.44-1.71)	0.749
Hospital length of stay of index hospitalization, in days, median (IQR)	3.0 (2.0-7.0)	2.0 (2.0-4.0)	10.0 (6.0-17.0)	≤0.001		
Follow-up since the index hospitalization, in days, median (IQR)	249.0 (152.0-438.5)	257.5 (153.2-423.0)	236.0 (151.0-453.0)	0.883		
Number of morbidities, in *n* (%)						
0	73 (36.6%)	67 (45.9%)	6 (11.3%)		^∗^	
1	63 (31.7%)	46 (31.5%)	17 (32.1%)		4.08 (1.40-13.64)	0.005
≥2	63 (31.7%)	33 (22.6%)	30 (56.6%)		9.96 (3.62-32.27)	≤0.001
Morbidities, in *n* (%)	126 (63.3%)	79 (54.1%)	47 (88.7%)		6.59 (2.59-20.04)^∗^	≤0.001
Asthma	72 (36.2%)	45 (30.8%)	27 (50.9%)		2.32 (1.16-4.66)	0.01
Neurologic diseases	41 (20.6%)	19 (13.0%)	22 (41.5%)		4.70 (2.14-10.47)	≤0.001
Gastrointestinal diseases	24 (12.1%)	7 (4.8%)	17 (32.1%)		9.24 (3.34-28.47)	≤0.001
Congenital or chromosomic abnormalities	22 (11.1%)	7 (4.8%)	15 (28.3%)		7.73 (2.74-24.13)	≤0.001
Heart failure	19 (9.5%)	9 (6.2%)	10 (18.9%)		3.51 (1.20-10.48)	0.01

^∗^Reference (1) does not have this morbidity. COVID-19: coronavirus disease, 2019; IQR: interquartile range; OR: odds ratio; 95% CI: 95% confidence interval.

## Data Availability

The data used to support the findings of this study are available from the corresponding author upon request.
